# Distinguished representation of identical mentions in bio-entity coreference resolution

**DOI:** 10.1186/s12911-022-01862-1

**Published:** 2022-04-30

**Authors:** Yufei Li, Xiangyu Zhou, Jie Ma, Xiaoyong Ma, Pengzhen Cheng, Tieliang Gong, Chen Li

**Affiliations:** 1 School of Computer Science and Technology, Xi’an Jiaotong University, Xi’an, 710049 Shaanxi China; 2 National Engineering Lab for Big Data Analytics, Xi’an Jiaotong University, Xi’an, 710049 Shaanxi China; 3 Shaanxi Province Key Laboratory of Satellite and Terrestrial Network Technology Research and Development, Xi’an Jiaotong University, Xi’an, 710049 Shaanxi China

**Keywords:** Coreference resolution, Mention detection, Neural network, Context-aware

## Abstract

**Background:**

Bio-entity Coreference Resolution (CR) is a vital task in biomedical text mining. An important issue in CR is the differential representation of identical mentions as their similar representations may make the coreference more puzzling. However, when extracting features, existing neural network-based models may bring additional noise to the distinction of identical mentions since they tend to get similar or even identical feature representations.

**Methods:**

We propose a context-aware feature attention model to distinguish similar or identical text units effectively for better resolving coreference. The new model can represent the identical mentions based on different contexts by adaptively exploiting features, which enables the model reduce the text noise and capture the semantic information effectively.

**Results:**

The experimental results show that the proposed model brings significant improvements on most of the baseline for coreference resolution and mention detection on the BioNLP dataset and CRAFT-CR dataset. The empirical studies further demonstrate its superior performance on the differential representation and coreferential link of identical mentions.

**Conclusions:**

Identical mentions impose difficulties on the current methods of Bio-entity coreference resolution. Thus, we propose the context-aware feature attention model to better distinguish identical mentions and achieve superior performance on both coreference resolution and mention detection, which will further improve the performance of the downstream tasks.

## Background

### Context and motivation

Bio-entity Coreference Resolution focuses on identifying the coreferential links in biomedical texts, which is a crucial task for artificial intelligence systems to be capable of fully understanding the biomedical texts, as significant entities are highly likely to be mentioned multiple times throughout the texts. Moreover, by improving the performance of several downstream tasks, including information extraction [1–3], entity linking [4], question answering [5], it can further break sentential boundaries and connects entities from texts, which is beneficial for both extracting complete bio-events and constructing bio-networks.

**Fig. 1:**
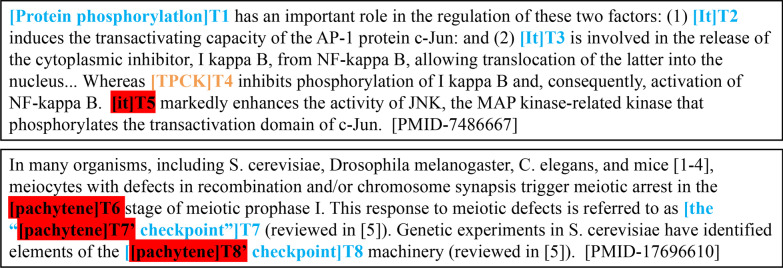
Example of linking errors of identical mentions affected by similar representations. The correct prediction is marked blue. The false link errors are highlighted in red. Correct annotations: {T1, T2, T3} in eg.1, {T7, T8} in eg.2

An important challenge in Bio-entity coreference resolution is identical mentions (see Task Section for the definition) as they tend to get similar or even identical representations, which makes the coreference more puzzling. This often leads to two types of wrong predictions: the false coreferential link among string matching mentions and the false local coreferential link among sub-string matching mentions. First, considering the first example as shown in Fig. 1, in previous neural network-based models, the undistinguished representation of string matching mentions: “it” T2, T3, T5 during feature extraction makes them close in feature vector space. This likely leads to the false prediction between T5 and T2, T3. Similarly, for the latter example, sub-string matching mentions: “pachytene” T6, “the “pachytene checkpoint”” T7 and “pachytene checkpoint” T8 overlap in “pachytene”. The undistinguished representation of the overlap likely causes the false local coreferential link between T6 and T7’, T8’.

Generally, the differential representation of identical mentions based on context is necessary as the above problem accounts for a large proportion of the coreference dataset. Different from general texts, biomedical texts contain many professional terms along with their synonyms, and most of the mentions are string matching or sub-string matching, which makes the problem more serious. So, in this paper, we focus on the distinguished representation of identical mentions in biomedical texts. Take protein coreference as an example, we make statistics on the identical mentions of the BioNLP dataset [6] and CRAFT-CR dataset [7]. First, BioNLP primarily annotates the coreferential links among protein/gene noun phrases, pronouns, and determiners. We first count the identical mentions in each document and find that documents containing identical mentions account for 42.9% of the whole in training dataset and 52% in the development dataset. However, approximately 20% of these identical mentions lack a coreferential link. In Table 1, we further analyze the POS (part of speech) tag of these identical mentions and their frequency (the number of times the identical mention appears in the document). The results show that these identical mentions are mostly determiners and wh-determiners, which is consistent with the annotation criteria of the dataset. Moreover, documents containing two identical mentions account for the largest proportion.

**Table 1:** Statistics of identical mentions on training and development set of BioNLP

	Frequency	NN/NNS/NP	PRP	WH-	IN	All
Train	2	49	66	80	107	302
	3	7	13	33	43	96
	> 3	14	12	9	28	63
	All	70	91	122	178	461
Dev	2	7	11	18	20	56
	3	1	5	11	7	24
	> 3	0	0	0	7	7
	All	8	16	29	34	87

The training and development sets have 800 and 150 documents, respectively. Each represents the number of documents containing identical mentions with different frequency of different POS tag.

Second, CRAFT-CR covers a wider range of coreferences and mainly focuses on noun phrases and events, we additionally make statistics on the number of clusters with identical mentions and find that about 65% clusters containing identical mentions. We further find that where nearly 52% of the entity clusters contain string matching mentions that account for more than half of the cluster’s total mentions. Consider that most of the coreferential links exist among nouns and noun phrases in CRAFT-CR, we make detailed statistics on whether there are string matching and sub-string matching between coreferential mention pairs in the Table 2. The results show that about 70% of the mention pairs have string matching or sub-string matching, which indicates that such large-scale coreferential annotations in CRAFT-CR cause the coreference system more likely to make wrong coreferential links between string matching mentions and wrong local coreferential links between sub-string matching mentions.

**Table 2:** Statistics on whether there are string matching and sub-string matching between coreferential mention pairs on CRAFT-CR

	String matching	Sub-string matching	Others
Train (%)	61.4	8.3	30.3
Dev (%)	58.2	7.0	34.8

Existing neural network-based models [7–10] have achieved reasonable good performance by applying the neural network with integrate domain specific information via pre-trained embeddings and bio-related features. However, these methods do not focus much on the distinction of identical mentions, which may further lead to wrong coreferential links as we mentioned above. Specifically, though context sensitive encoding networks (such as Bi-LSTM and RNN) can distinguish identical mentions based on the contexts. But, by using one-hot mapping for feature extraction, identical mentions often get similar or even the same feature representation. This means they are close in the feature vector space, which will bring additional noise to their distinction. In this paper, our goal is to distinguished represent these identical text units based on context, so that shorten the distance between identical text units with similar contexts and increase the distance between those with different contexts in feature space. Thus, we propose a general context-aware feature attention mechanism that adaptively learns the importance of each feature based on contexts, so as to re-encodes the feature during the feature fusion. Thus, the context based re-encoded features can reduce the noise brought by the similar representation of identical mentions, so as to better distinguish them.

The experiments are conducted by fusing the feature attention mechanism on several neural network-based methods [9, 10]. The proposed model is evaluated on the BioNLP Protein Coreference dataset [6] and CRAFT-CR dataset [7]. The experimental results show that the proposed model brings improvements on most the baselines. Specifically, for [9], it brings 2.0% F1 on BioNLP and 0.5% F1 on CRAFT, and for [10], it brings 0.3% F1 on CRAFT, which achieves the state-of-the-art performance. Additional experiments on mention detection also achieve the state-of-the-art performance with 81.1% F1 on BioNLP and 71.5% F1 on CRAFT. The results reveal the effectiveness of our model in extracting the semantic information and reducing the text noise. Furthermore, empirical studies on identical mention coreference demonstrate that the feature attention mechanism aids in distinguishing identical mentions based on different contexts by reducing the noise.

### Related work

In bio-entity coreference resolution tasks, words referring to each other are called mentions, while a mention can either be a common noun, a proper noun, or a pronoun. Taking the first example in Figure 1, a coreference system partitions the mentions into two coreference chains: (“Protein phosphorylation”, “it”, “it”), and (“TPCK”, “it”).

In recent years, the task has attracted researchers’ attention because of its great potential in biological research. The BioNLP protein Coreference dataset [6] and the CRAFT-CR dataset [7] are two typical datasets for bio-entity coreference resolution. The former are abstracts extracted from PubMed and primarily focuses on coreference among protein/gene. The latter consists of full papers extracted from PMC, covering a broader range of coreferences. Previous work on these two datasets can be categorized into three classes. (1) rule and feature-based models [8, 11, 12] which heavily rely on syntactic parsers to extract manually crafted features and rules, (2) hybrid models [13, 14], which combine rule-based and machine learningbased methods for biomedical coreference resolution, (3) neural network-based models [7–10], which use deep learning or neural networks to make coreferential links automatically through domain-specific information integration, including pre-trained embeddings and some biomedical features.

Generally, the above work is summarized in Table 3. Our work is most closely related to the work of [9, 10], while we focus on the problem that previous neural network-based methods may cause noise for the distinction of identical mentions during feature extraction, since they often get similar or even the same feature representation. This further mislead to make coreferential mistakes.

**Table 3:** Coreference resolution performance comparison by the average F1 value

	Dev-F1	Test-F1	Feature-based Rule-based	Hybrid	Neural
*BioNLP*					
[13]	62.4	60.9		√	
[14]	68.6	/		√	
[11]	63.9	48.1	√		
[12]	67.5	/	√		
[8]	72.2	62.0	√		√
[9]	63.4	51.2			√
[10]	65.6	69.5			√
*CRAFT*					
[7]	45.5	46.4			√
[9]	33.9	36.0			√
[10]	/	57.0			√

All the models are evaluated on the platforms provided by the task organizers

## Methods

### Task

In an end-to-end coreference resolution system, the input is a document *D* with *T* words, and the output is a set of mention clusters. Let *N* be the number of possible text spans in *D*. We consider all possible spans up to a predefined maximum width. *START*(*i*) and *END*(*i*) are the start and end indices of a span *i* in *D* respectively. For each span *i* the system needs to assign an antecedent ${a}_{i}\in \{\varepsilon ,1,\dots ,i-1\}$ from all preceding spans or a dummy antecedent $\varepsilon$. The dummy antecedent represents two cases: (1) the span *i* is not a mention, or (2) the span *i* is a mention but not coreferential with any previous span. Finally, all spans that are connected by a set of antecedent predictions are grouped.

The formal definition of identical mention is as follows. Suppose the N mentions in a document D are denoted as $\mathrm{M}=\{{m}_{1},{m}_{2},\dots {m}_{N}\}$. The identical mentions are defined by: $\mathrm{M}=\{{m}_{i}|\exists {m}_{j}={m}_{i}\mathrm{ and }{m}_{j}\in \mathrm{M and j}\ne \mathrm{i}$. For each identical mention *m*_*i*_, we define its frequency as the number of times that this mention appears in the document.

### Baseline model

In this section, we briefly describe the baseline model [15] which is the basic model of most of the neural network-based Bio-entity coreference systems [7, 9, 10].

#### Span representation

Assuming vector representation of a sentence with *L* words as $\{{x}_{1},{x}_{2},\dots ,{x}_{L}\}$, while ${x}_{t}$ denotes the concatenation of fixed pre-trained word embeddings and CNN (convolutional neural network) character embeddings [16] for *t*th word. The Bi-LSTMs (Bidirectional long short-term memory) [17] are used to encode each ${x}_{t}$.

Then, the model uses the attention mechanism [18] over words in each span to learn a task-specific notion of headedness, and the final representation ${g}_{i}$ of span *i* is produced by:

1 $${g}_{i}=[{x}_{START\left(i\right)}^{*},{x}_{END\left(i\right)}^{*}, \widehat{{x}_{i}},\varphi (i)]$$

where ${x}_{START\left(i\right)}^{*}$ and ${x}_{END\left(i\right)}^{*}$ are the outputs of Bi-LSTM corresponding to the first and last word of the span *i*. $\widehat{{x}_{i}}$ is the head embedding encoded by the head attention mechanism. $\varphi (i)$ is the feature vectors.

#### Scoring

The scoring functions: mention score ${s}_{m}$ and antecedent score ${s}_{a}$ take the span representations as input. All the spans are ranked based on their mention scores ${s}_{m}$. The coarse antecedent score which denotes whether span *i* is coreferential with span *j* is calculated as ${s}_{a}$:

2 $${s}_{m}\left(i\right)={w}_{m}\bullet {FFNN}_{m}({g}_{i})$$

3 $${s}_{a}\left(i,j\right)={w}_{\alpha }\bullet {FFNN}_{\alpha }([{g}_{i},{g}_{j},{g}_{i}^\circ {g}_{j},\varphi (i,j)])$$

where ${w}_{m}$ and ${w}_{\alpha }$ are the weight matrix, ◦ denotes element-wise multiplication, $FFNN$ is the feed-forward neural network, and $\varphi (i,j)$ is the pair-wise features encoding the distance between the two spans.

## Feature attention

### Model structure

To reduce the noise brought by features and distinguish the identical mentions effectively, we propose a context-aware attention mechanism called Feature Attention to adaptively exploit features based on context.

**Fig. 2:**
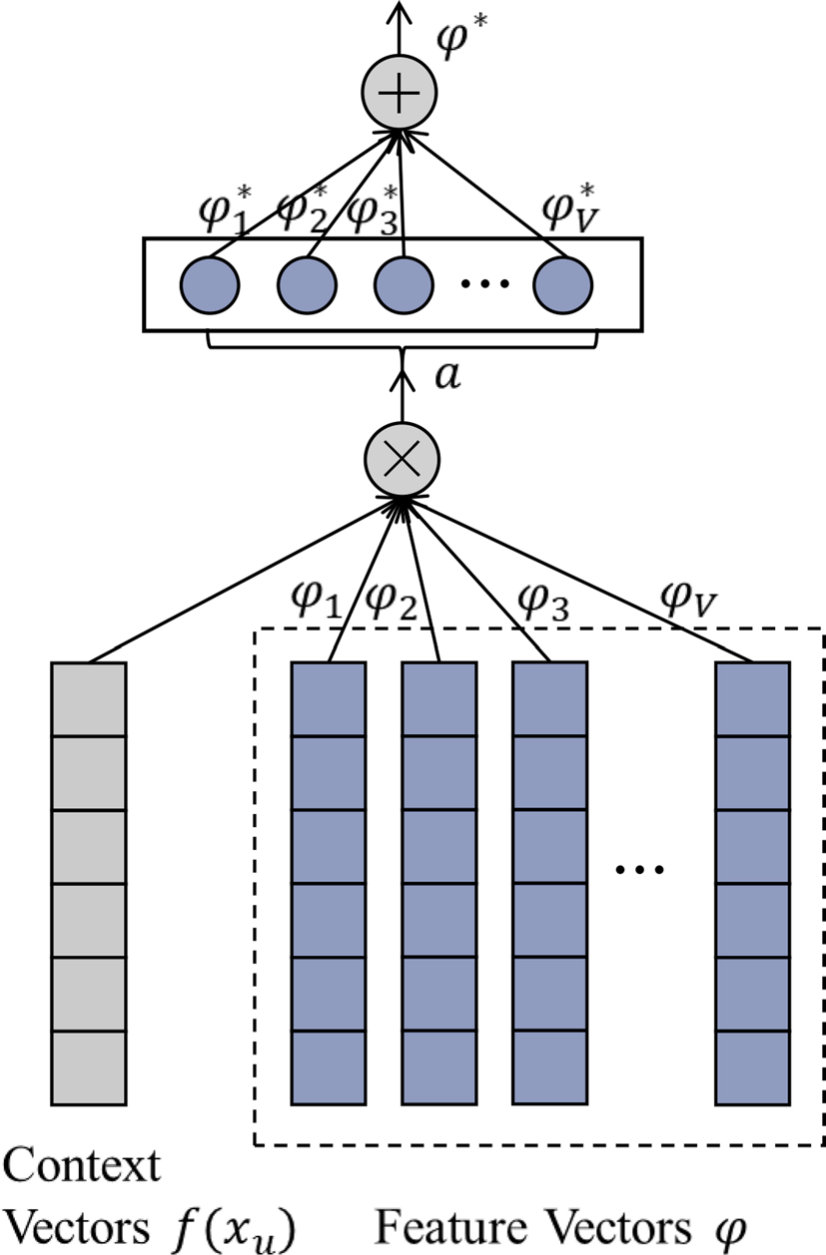
The Feature Attention model. The model learns to weigh each feature based on contexts

As shown in Fig. 2, we use a general attention mechanism that learns the importance or weight of each feature based on contexts. Suppose the initial feature vectors is $\mathrm{\varphi }=[{\varphi }_{1},{\varphi }_{2},\dots {\varphi }_{V}]$, where ${\varphi }_{j}$ indicates the *j* − *th* feature and ${x}_{u}^{*}$ is the contexts vectors generated by Bi-LSTM for span *u* (here we use the average of the context representation of each word in the span). Then the model learns the weight of each feature based on the contexts, and generate new feature vectors ${\varphi }^{*}$:

4 $${a}_{j}={w}_{a}\bullet FFNN({\varphi }_{j}f({x}_{u}^{*}))$$

5 $${a}_{j,u}=\frac{\mathrm{exp}({a}_{j})}{{\sum }_{v=1}^{V}\mathrm{exp}({a}_{v})}$$

6 $${\varphi }^{*}={\oplus }_{j=1}^{V}{a}_{j,u}\bullet {\varphi }_{j}$$

where ⊕ is the concatenation operation and $f({x}_{u}^{*})$ is a linear function to map ${x}_{u}^{*}$ to the same dimension with the feature vector. ${a}_{j,u}$ is the weight of each feature based on the contexts and ${\varphi }^{*}$ is the new reweighed feature vectors.

### Span feature attention

To use features adaptively, we apply the Feature Attention mechanism to the span features: span width, grammatical number, and Metamap entity tags.

**Fig. 3:**
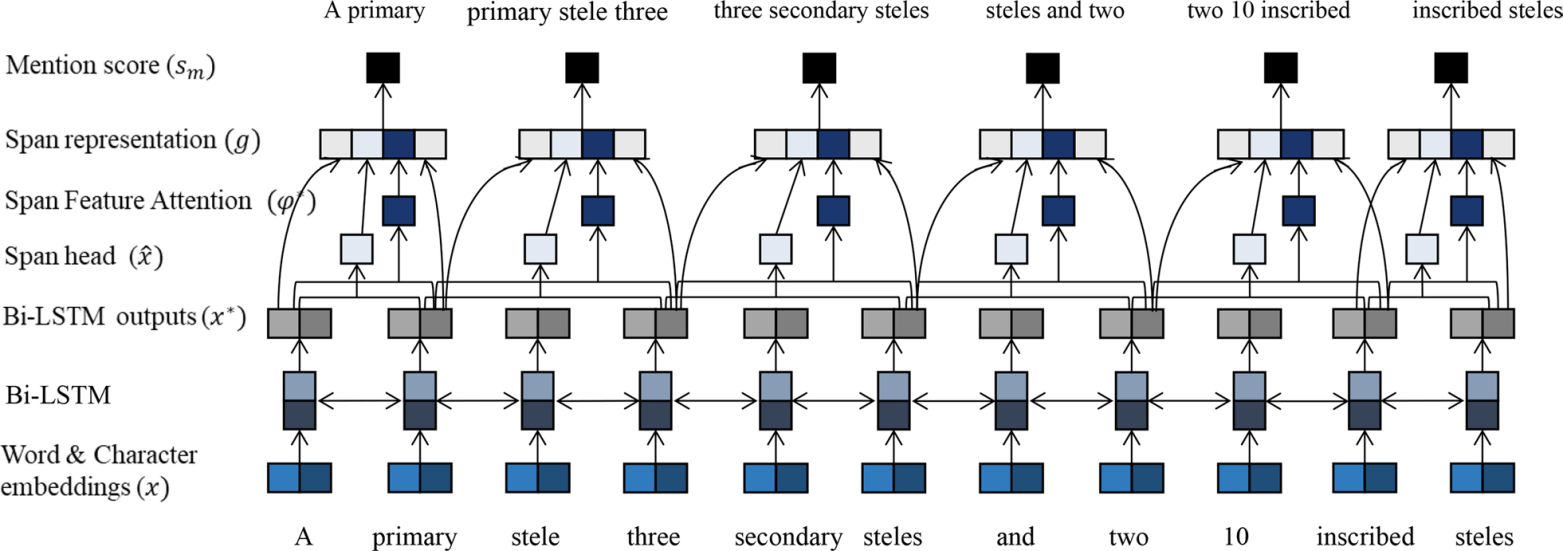
The model of computing the span embedding representations

As shown in Fig. 3, a new context-aware feature vector ${\varphi }^{*}$ is generated by the Feature Attention method and the new span features are applied to update the span representation, where ${x}_{u}^{*}$ is the contexts vectors generated by Bi-LSTM for span *i* and FA is the Feature Attention mechanism:

7 $${\varphi }^{*}\left(i\right)=FA(\varphi \left(i\right),{x}_{u}^{*})$$

8 $${g}_{i}=[{x}_{START\left(i\right)}^{*},{x}_{END\left(i\right)}^{*}, \widehat{{x}_{i}},{\varphi }^{*}\left(i\right)$$

### Coreference Score

The final coreference score of span *i* and *j* shows that (1) whether span *i* is a mention, (2) whether span *j* is a mention and (3) whether *j* is an antecedent of *i*:

9 $$\mathrm{s}\left(\mathrm{i},\mathrm{j}\right)=\left\{\begin{array}{c}0, j=\varepsilon \\ {s}_{m}\left(i\right)+{s}_{m}\left(j\right)+{s}_{a}\left(i,j\right)+{s}_{c}\left(i,j\right), j\ne \varepsilon \end{array}\right.$$

10 $${s}_{c}\left(i,j\right)={g}_{i}^{T}{w}_{c}{g}_{j}$$

where ${s}_{m}\left(i\right)$ is the mention score derived by Eq. (2), ${s}_{a}\left(i,j\right)$ is the antecedent score derived by Eq. (3), ${s}_{c}\left(i,j\right)$ is a rough sketch of likely antecedents and ${w}_{c}$ is a learned weight matrix.

## Experiments

### Dataset and baseline

The experiments are performed on the BioNLP Protein coreference dataset [6] and CRAFT-CR dataset [7]. The BioNLP Protein Coreference dataset consists of 1210 PubMed abstracts and mainly focuses on protein/gene coreference. In CRAFT, there are 97 full papers extracted from PMC, covering a broader range of coreferences. For BioNLP, we use the scorer^1^ provided by the organizers to make a fair comparison with the previous work. For CRAFT-CR, the dataset is divided into three subsets in a ratio of 6:1:3 for training, development, and test. We evaluate it on the platform^2^ provided by the task organizers.

To show the effectiveness of the proposed method, we conduct the experiments by fusing our Feature Attention mechanism on several neural network-based methods. As most previous neural network-based methods’ codes are not open accessed [8], we only use the following open accessed ones [9, 10]. Because [9] is based on the old version [19] of the neural system [15], we modify it with the new one [15].

- BERTfilter [7]: The system provides: (1) a filter of noisy mentions based on parse trees.(2) an integration of language model BERT.
- Lee2018 [8]: The system aprovieds (1) a rule-based method (2) a machine learning-based method using LSTM network.
- Bioe2e [9]: The system applies a state-of-the-art general system [15] with domain-specific features for biomedical text.
- KE-LSTM [10]: The system proposes a knowledge enhanced LSTM to better resolve bio-entity coreference.
- Simple [11]: The system develops a rule-based system with simple modules derived from available systems.
- Bio-SCoRes [12]: The system presents a novel, highly flexible architecture and provided a set of strong, linguistically-based baseline methods.
- Hybrid [13]: The system proposes a hybrid approach that combines both rule-based and learningbased method.

### Hyperparameters

We follow the same hyperparameters as in [15]. For input words, we use (1) GloVe [20] word embeddings pre-trained on Pubmed with a window size of 2; (2) ELMo embeddings we trained on the PubMed with ELMo [21]; (3) BERT embeddings trained by the language model BERT [22] on general domains. For headword, we use GloVe [20] word embeddings pre-trained on Pubmed with a window size of 2. We only consider 50 antecedents and the maximum span width is 30 for BioNLP and 10 for CRAFT-CR. The ratio of top span is set up to 0.7 and 0.35 for BioNLP and CRAFT-CR, respectively. The model is trained up to 70 epochs with early stopping. Instead of Universal Sentence Encoder [23], we use Bi-LSTM to encode sentences and the window size is set up to 10. This is because (1) compared with Bi-LSTM, USE requires a higher amount of computation. Due to the limited computing resources, we have to reduce the values of hyperparameters: maximum span width and ratio of top span. This will limit the performance. (2) We have used pre-trained BERT embeddings, which overlaps with USE to some extent. To show the validity of the Feature Attention mechanism, we consider the following experiments:

- BioNeu: modify [9] with the new neural network system [15].
- BioNeu-feature: BioNeu without span features (span width, grammatical number, and Metamap entity tags).
- BioNeu + SFA: BioNeu with SFA (span feature attention) mechanism.
- KE-LSTM: a knowledge enhanced bio-entity coreference system [10].
- KE-LSTM-feature: KE-LSTM [10] without features (span width, grammatical number, and Metamap entity tags).
- KE-LSTM + SFA: KE-LSTM [10] with SFA (span feature attention) mechanism.

## Results

### Evaluation of coreference

Tables 4 and 5 show the performance comparison on the development and test set of BioNLP and CRAFT-CR, respectively. We respectively modify the two baselines by removing the features (-feature) and proposing SFA (+SFA). BioNeu is the modified one of [9] as we mentioned in section Dataset and Baseline. From the experimental results, we have the following observations. First, the results display that the CRAFT-CR corpus is more challenging than the BioNLP dataset as the scores are always lower on the CRAFT-CR dataset. Second, compared with BioNeu and KE-LSTM [10], the performance is reduced when features are removed. This indicates that domain-related features do help in the domain task. However, these features will bring noise to identical mentions which is further proved and discussed in section Identical Mention Coreference Evaluation.

**Table 4:** The performance of protein coreference resolution with different models on two evaluation datasets of BioNLP

	Dev			Test		
	P	R	F1	P	R	F1
Hybrid	59.9	**77.1**	67.4	55.6	67.2	60.9
Simple	63.4	64.4	63.9	46.3	50.0	48.1
Bio-SCoRes	72.4	63.2	67.5	/	/	/
Lee2018-rule	68.8	76.0	**72.2**	60.2	63.8	62.0
lee2018-neural	60.4	61.9	61.2	54.9	58.0	56.4
Bioe2e	71.7	56.7	63.1	55.6	47.5	51.2
BioNeu	**77.1**	61.9	68.7	71.5	60.9	65.8
BioNeu-feature	75.5	65.8	70.4	69.5	60.2	64.5
BioNeu + SFA	73.0	65.3	69.0	**72.3**	61.6	66.5
KE-LSTM	68.1	63.4	65.6	69.6	**69.4**	**69.5**
KE-LSTM-feature	74.4	64.8	69.3	62.8	61.2	62.0
KE-LSTM + SFA	70.8	68.3	69.6	69.5	68.2	68.8

The maximum value is in bold

**Table 5:** F1 scores of coreference on CRAFT test set in comparison with some baselines

System	B^3^	BLANC	CEAFE	CEAFM	LEA	MUC	Ave
E2E_MetaMap	36.4	46.5	33.1	41.0	32.4	51.8	40.2
BERTfilter	44.0	48.9	39.8	49.0	40.0	57.0	46.4
BioNeu	45.0	55.4	36.1	49.8	41.8	55.1	47.2
BioNeu-feature	45.3	53.2	36.5	49.4	42.1	56.1	47.1
BioNeu + SFA	45.1	56.2	37.0	49.7	42.0	56.3	47.7
KE-LSTM	54.9	63.1	48.6	59.4	51.3	64.5	57.0
KE-LSTM-feature	54.5	62.2	48.1	59.2	51.4	64.5	56.6
KE-LSTM + SFA	**55.0**	**63.6**	**49.5**	**59.4**	**51.7**	**64.6**	**57.3**

E2E_MetaMap and BERTfilter are the baselines in [7]

The maximum value is in bold

Last, compared with BioNeu, the Feature Attention mechanism brings improvements on all the baselines. Specifically, for BioNeu, it brings 2.0% F1 on BioNLP and 0.5% F1 on CRAFT, and for KELSTM [10], it brings 0.3% F1 on CRAFT. Though KE-LSTM + SFA gains performance on the development of BioNOLP, it shows limitation on the test. The results suggest that the distinction of mentions based on contexts is vital for effectively resolving coreference. In this case, context-aware attention models will assist in achieving this goal and making accurate predictions. For the limitation of KE-LSTM + SFA on BioNLP, it may because for the most coreferential annotations that exist between noun or noun phrase and determiners, the introduction of SFA intensifies the difference of features between the two mentions. And this brings noise to the system. In general, we notice that our model performs much better precision than competitors on the basis of ensuring recall, indicating that after the distinct representation of mentions based on context, the noise brought by the representation of similar or identical mentions is reduced. Besides, we also find that the proposed model has a stronger generalization ability than competitors on the BioNLP.

### Mention detection subtask

To further understand the utility of the Feature Attention mechanism for mention detection subtask, we list the mention detection performance of the two datasets in Table 6. For both the two datasets, compared with Bioneu and KE-LSTM [10], the performance is significantly increased when SFA is introduced to the baselines. Moreover, the SFA model indeed performs much better in the recall scores. This indicates that, in the baseline model, where there is a span not predicated as a mention, the other identical spans will likely not be detected as mentions due to their similar representations. However, in the SFA model, such false-negative errors are decreased, having benefited from the Feature Attention mechanism that reweighs the features to distinguish identical spans with different representations based on different contexts.

**Table 6:** The performance of mention detection with different models on two datasets

BioNLP	P	R	F1
Bioe2e	82.0	66.3	73.3
BioNeu	**84.1**	73.1	78.2
BioNeu + SFA	83.4	76.1	79.6
KE-LSTM	78.0	84.1	80.9
KE-LSTM + SFA	78.2	**84.3**	**81.1**
CRAFT	P	R	F1
E2E_MetaMap	67.1	52.7	59.0
BERTfilter	73.1	57.8	64.5
BioNeu	81.6	49.6	61.7
BioNeu + SFA	**83.4**	49.4	62.0
KE-LSTM	79.3	63.1	70.3
KE-LSTM + SFA	78.8	**65.6**	**71.5**

E2E_MetaMap and BERTfilter are the baselines in [7]. Bioe2e is the baseline in [9], and KE-LSTM is the baseline in [10]

The maximum value is in bold

## Discussion

### Identical mention coreference evaluation

To demonstrate the efficacy of the Feature Attention mechanism in the distinguished representation of identical mentions and their coreference resolution, we make statistics on the performance of identical mention coreference among baseline, baseline-feature, and baseline+SFA. For BioNLP, Table 7 shows the performance of different models on identical mention coreference with different POS tags. The results display that compared with the two baselines (Bioneu and KE-LSTM [10]), the performance of identical mention coreference is improved when features are removed, and the superior is more obvious on precision. This is consistent with our previous analysis that features integrated in neural network-based model will bring noise to identical mentions, because identical mentions often have similar or even the same feature representation, which may further lead to wrong coreferential links. Moreover, when SFA mechanism is introduced in the two baselines, such noise brought by features is reduced, thus improving the performance of identical mention coreference. Last, for different POS tags, we find that the SFA is more effective on NN/NNS/NP (noun or noun phrase), which may be because that features play a more vital role in the semantic representation of such noun or noun phrase. Thus SFA helps the model to better distinguish such noun or noun phrase by further distinguishing their features.

**Table 7:** The coreference performance of different models on identical mentions with different POS tags on BioNLP dataset

	NN/NNS/NP			PRP			WH			IN		
	P	R	F1	P	R	F1	P	R	F1	P	R	F1
BioNeu	62.5	17.8	27.7	39.0	37.8	38.4	68.3	73.0	70.6	65.6	64.3	64.9
BioNeu-feature	42.9	21.4	28.5	46.3	41.3	43.6	69.1	75.7	72.2	70.1	74.2	72.1
BioNeu + SFA	52.9	32.1	40.0	45.7	34.8	39.5	72.0	73.0	72.5	71.6	72.3	71.9
KE-LSTM	66.7	21.4	32.4	45.0	39.1	41.8	63.5	73.0	67.9	67.6	68.3	68.0
KE-LSTM-feature	75.0	21.4	33.3	47.0	34.9	40.1	71.8	75.6	73.6	72.0	76.2	74.0
KE-LSTM + SFA	92.3	80.0	85.7	47.5	50.0	48.7	66.3	77.5	71.5	60.4	63.4	61.9

KE-LSTM is the baseline in [10]

In Table 8, we compare the coreference performance of different models on three types of mention pairs on CRAFT-CR: String matching, Sub-string matching and Others. Among them, the performance of identical texts units representation will directly affect the coreference performance of String matching mention-pairs, followed by Sub-string matching ones. First, the results show that compared with the two baseline models (BioNeu, KE-LSTM [10]), the performance of string or sub-string matching mention pairs is improved when features are removed, and for those mention pairs that do not have string or sub-string matching, the performance is limited. This demonstrates that features integrated in neural network-based model help in the coreferential prediction of mention pairs with larger differences but will bring noise to identical mentions. Furthermore, the performance is improved when SFA mechanism is introduced to the baselines, especially on precision. Such superiority indicates that the Feature Attention mechanism does help to reduce the noise brought by features and distinguish the identical mentions based on context, which provides further help in coreference. Besides, SFA is more powerful on string matching mention pairs. This is consistent with the superiority of SFA in distinguishing identical texts units.

**Table 8:** The coreference performance of mention pairs on CRAFT-CR in three cases

	String match			Sub-string match			Others		
	P	R	F1	P	R	F1	P	R	F1
BioNeu	62.5	71.5	66.7	42.6	27.9	33.7	44.1	29.6	35.4
BioNeu-feature	68.4	68.9	68.6	51.4	32.9	40.1	31.9	29.9	30.9
BioNeu + SFA	68.8	67.8	68.3	44.2	28.2	34.4	47.5	29.3	36.3
KE-LSTM	64.8	83.6	73.1	39.8	35.3	37.4	27.2	34.8	30.5
KE-LSTM-feature	66.6	81.5	73.3	47.1	34.3	39.7	24.0	37.4	29.3
KE-LSTM + SFA	70.2	82.8	76.0	41.4	36.9	39.1	29.3	32.8	30.9

KE-LSTM is the baseline in [10]

### Remaining problems and future work

**Fig. 4:**
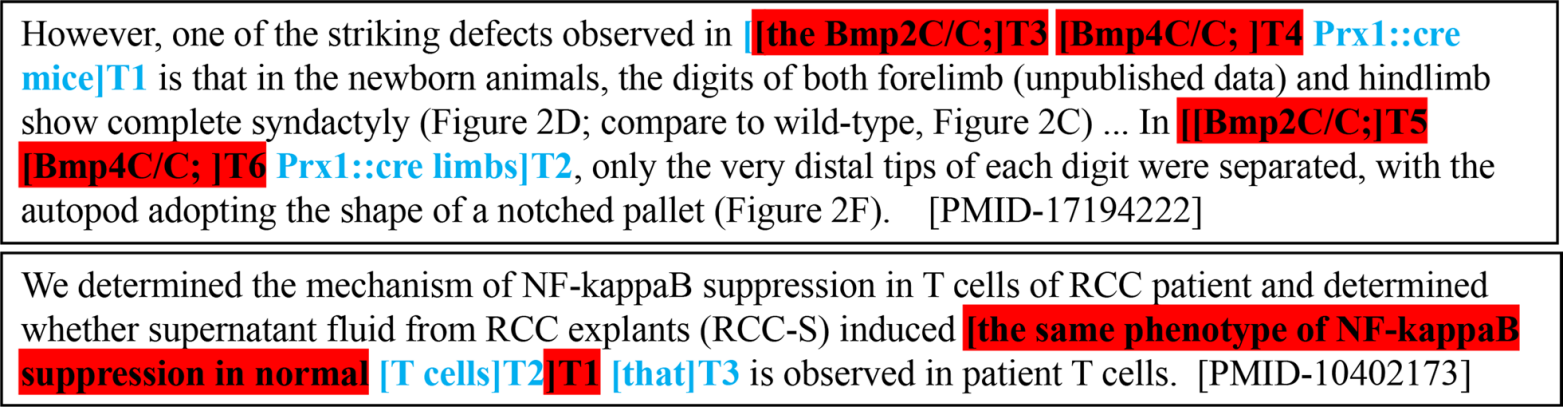
Examples of remaining problems. The correct prediction is marked blue. The spurious link errors are highlighted in red

Finally, we find that the proposed model has some limitations. First, from the results of identical mention coreference evaluation on CRAFT-CR, we find that the model is limited when there is no string or sub-string matching between mention pairs. According to our former statistics, this may be because most of the coreferential annotations in CRAFT-CR exist between string or sub-string matching mention pairs, which cause the model to be trained to pay more attention to the links between identical mentions. Furthermore, on CRAFT-CR, we also find that when the mention’s length (number of tokens within the mention) is much long and sub-string matching, it is likely to make the local coreferential link. Considering the first example in Figure 4, there are two long mentions: “the Bmp2C/C; Bmp4C/C; Prx1::cre mice” T1 and “the Bmp2C/C; Bmp4C/C; Prx1::cre limbs” T2. Due to their long length and sub-string matching, the model tends to make local predictions between “the Bmp2C/C’;” T3 and “the Bmp2C/C’;” T4, “Bmp4C/C;” T5 and “Bmp4C/C;” T6. Third, for BioNLP, we find that when there are several syntactic relations in the sentence, such as preposition-object relation, coordinate relation, subordination relation, etc. it is hard to predict the coreference. As shown in the second example in Figure 4, the model makes the false link between “the same phenotype of NF-kappaB suppression in normal T cells” T1 and “that” T3, because of the complex relations among spans: “the same phenotype”, “NF-kappaB suppression”, and “normal T cells”.

Therefore, there are several potential improvements to our model as future work. First, for the false link and the local link caused by the emphasis of coreferential links among string matching mentions in the dataset, maybe we can balance the dataset by resampling or refining the loss function. Furthermore, considering that this approach is still limited when the syntactic relation is complex, we expect to utilize the syntactic information with the help of dependency trees.

### Case study

**Fig. 5:**
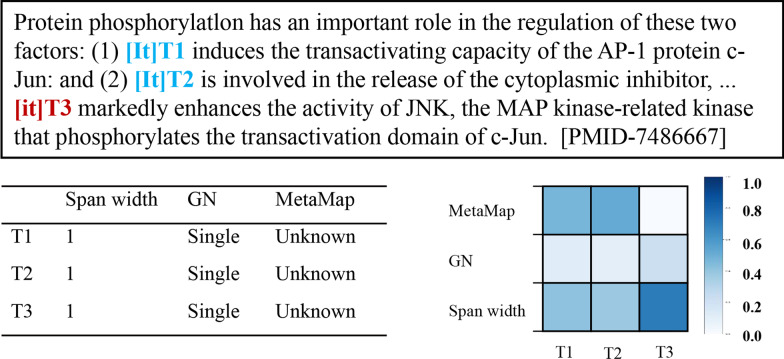
The visualization of mentions’ features and their attention weights in the first example. GN means grammatical numbers. Each column shows the attention weights of all the features of the span

**Fig. 6:**
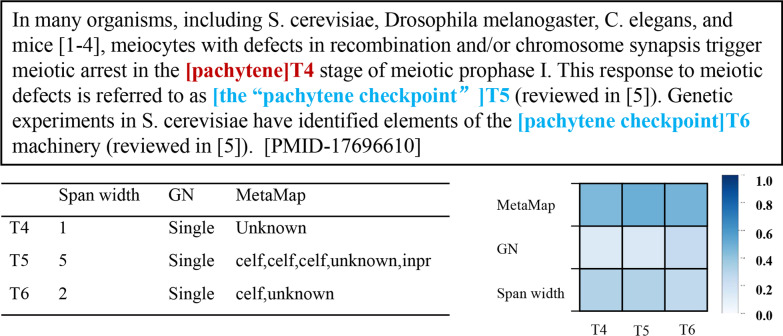
The visualization of mentions’ features and their attention weights in the second example. GN means grammatical numbers. Each column shows the attention weights of all the features of the span. “celf” means Cell Function comd. “inpr” means intellectual product

To gain further insight into how identical terms’ representations can be distinguished by the attention mechanism, we take the former case in Figure 1 as an example to investigate the Feature Attention weights. We list the value of span features (span width, grammatical number, and Metamap entity tags) and visualize the attention weights of them in Figures 5 and 6, where correct coreferential predictions are marked blue. In Figure 5, for those three “it”: T1, T2 and T3, they have the same feature value. While, the attention weights show that there is difference between T3 and the last two. The first two “it” that are coreferential (T1 and T2) gain similar weights for the three features, where the weight of Metamap is the highest, followed by span width, and finally the grammatical number. However, T3 has the different span weights with the descending order of span width, GN, and MetaMap. This displays that identical mentions with the same features will have different feature attention weights depending on their contexts through the span Feature Attention, thus benefits the model in distinguishing identical mentions and avoiding wrong links.

In Fig. 6, for the three mentions: “pachytene” T4, “the “pachytene checkpoint”” T5 and “pachytene checkpoint” T6, we can find there are some differences between T4 and the last two coreferential mentions, The Meatamp feature of T5 and T6 overlap in “celf”, that is, Cell Function comd. The span width feature of T5 and T6 is much longer than T4. Based on contexts, the Feature Attention mechanism helps the model distinguish T4 from the other two mentions by increasing the weight of MetaMap and span width. This further avoids the wrong local links.

**Fig. 7:**
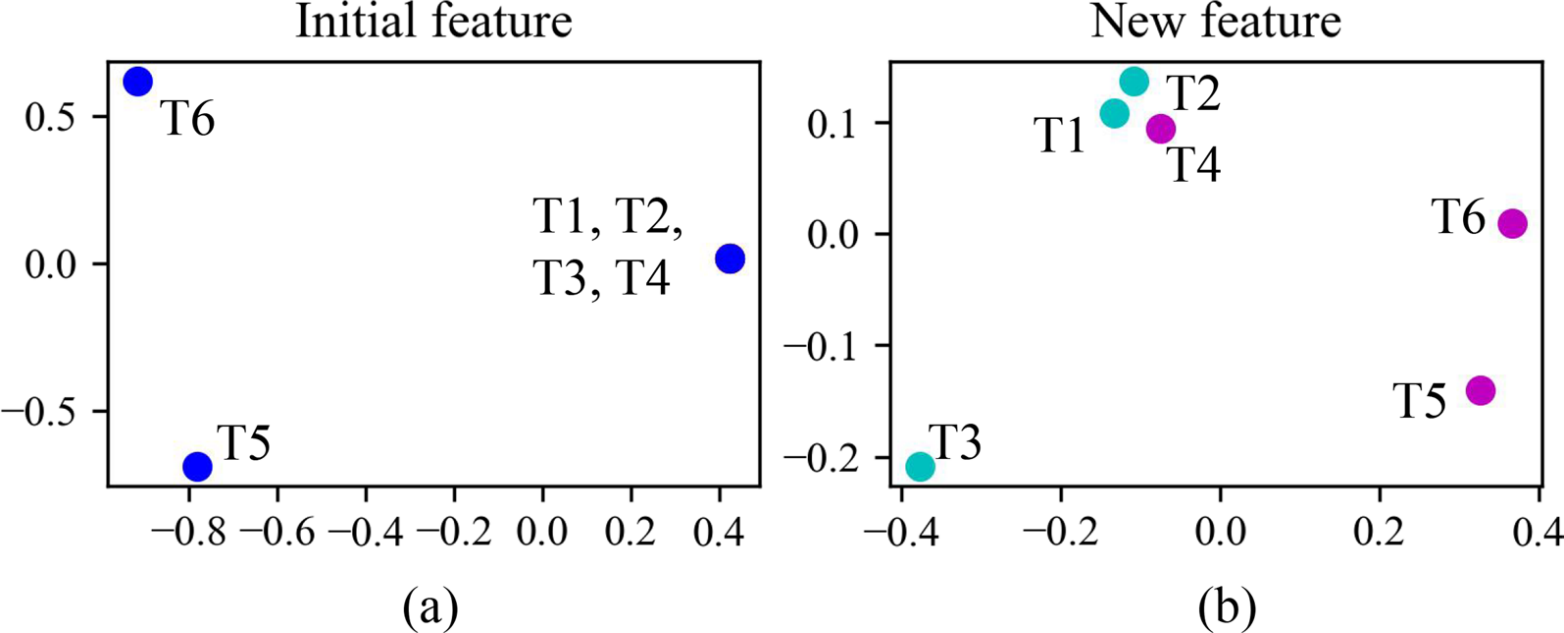
The reduced 2-dimensional feature representations before and after using SFA. We use PCA for dimensionality reduction. **a** The initial feature representations before using SFA, and **b** the new feature representations after using SFA

To show how the proposed model performs on the distinguished representation of identical mentions and their coreference resolution, for the above T1-T6, we use Principal Component Analysis (PCA) to reduce the dimension of their feature representation to two. The reduced 2-dimensional feature representations before and after using SFA are compared in Fig. 7, where (a) is the initial feature representations before using SFA, and (b) is the new feature representations after using SFA. In Fig. 7a, T1–T4 have the same feature representation, since they have the same feature (1, Single, unknown). In Fig. 7b, after using SFA, the coreferential mention pairs T1–T2, T5–T6 are close in the feature space, while T3, T4 are far from them. This will make the coreferential predictions easier.

## Conclusion

Identical mentions impose difficulties on the current methods of Bio-entity coreference resolution as they tend to get similar or even identical representations. This problem may directly lead to wrong predictions. In the paper, we focus on this issue and distinguish identical mentions by developing a context-aware feature attention model. We apply the attention mechanism in the process of span representation to adaptively exploit features and represent identical mentions considering different contexts. The results show that our model with the Feature Attention mechanism performs reasonably well in Bio-entity coreference resolution. The performance is supported on the BioNLP Protein Coreference dataset and CRAFT-CR dataset. Moreover, as our model learns to distinguish identical mentions more effectively, it achieves superior performance on the identical mention coreferece.

## Data Availability

The datasets used in this study are publicly available at the official website of BioNLP Shared Task 2011: https://sites.google.com/site/bionlpst/ and the CRAFT-CR dataset: https://github.com/UCDenver-ccp/CRAFT.

## Abbreviations

CR: Coreferece resolution
POS: Part of speech
RNN: Recurrent neural network
CNN: Convolutional neural network
Bi-LSTM: Bidirectional long short-term memory
GN: Grammatical number
USE: Universal sentence encoder
PCA: Principal Component Analysis

## Footnotes

1 http://bionlp-st.dbcls.jp/CO/eval-test/

2 https://github.com/UCDenver-ccp/craft-sharedtasks
